# Robot-assisted integrated cognitive-motor rehabilitation in patients with chronic stroke: a randomized controlled trial

**DOI:** 10.3389/fneur.2026.1866135

**Published:** 2026-08-13

**Authors:** Seungwoo Cha, Jiyeon Lee, Hyojin Jeon, Min Ho Chun

**Affiliations:** 1 Department of Rehabilitation Medicine, Asan Medical Center, University of Ulsan College of Medicine, Seoul, Republic of Korea; 2 Asan Institute for Life Sciences, Asan Medical Center, University of Ulsan College of Medicine, Seoul, Republic of Korea

**Keywords:** cognitive dysfunction, dual-task training, neurorehabilitation, robotic rehabilitation, stroke, upper extremity

## Abstract

**Background:**

Cognitive and motor impairments are major determinants of functional independence after stroke. While robot-assisted training is effective for motor recovery, its impact on cognitive function through integrated cognitive–motor training remains less explored. This study evaluated the effects of an end-effector robot-assisted integrated cognitive–motor rehabilitation program on global cognitive function and upper limb motor outcomes in patients with chronic stroke.

**Methods:**

In this single-center, randomized, assessor-blinded exploratory trial, 30 patients with stroke were randomized into an intervention group (*n* = 15) or a control group (*n* = 15). The intervention group received robot-assisted cognitive–motor training using the Rebless Planar system (30 min/session, 10 sessions over 3 weeks), which integrated goal-directed reaching with tasks involving attention, memory, and executive function. The control group received time- and dose-matched conventional paper-based cognitive rehabilitation. Primary outcomes were the Korean Mini-Mental State Examination (MMSE) and the Korean Montreal Cognitive Assessment (MoCA). Secondary outcomes included CERAD subdomains, Fugl–Meyer Assessment of the Upper Extremity (FMA-UE), and the Modified Barthel Index (MBI). This study was registered with the Korean Clinical Research Information Service (KCT0010564).

**Results:**

Both groups exhibited significant within-group improvements in MMSE and MoCA, with no statistically significant between-group differences. For secondary CERAD subdomains, observed within-group gains did not maintain statistically significant between-group differences after False Discovery Rate adjustment. However, upper extremity motor recovery was significantly superior in the intervention group (FMA-UE 33.6 to 37.5) compared to controls (25.7 to 25.9) (*p* = 0.012, Cohen’s *d* = 0.98).

**Conclusion:**

Robot-assisted integrated cognitive–motor rehabilitation using an end-effector system is feasible and safe in chronic stroke patients. While both robotic and conventional cognitive rehabilitation led to comparable improvements in global cognition, the robot-assisted group demonstrated superior upper limb motor recovery. These findings suggest that integrated dual-task training is a promising strategy for post-stroke motor recovery, though its incremental cognitive benefit over conventional therapy requires further investigation in larger trials.

**Clinical trail registration:**

This study was registered with the Korean Clinical Research Information Service (KCT0010564).

## Introduction

Stroke is a leading cause of long-term disability worldwide, frequently resulting in persistent motor and cognitive impairments that limit functional independence and quality of life (1). Upper extremity dysfunction remains one of the most disabling sequelae, and conventional rehabilitation often fails to provide sufficient intensity and task-specific repetition to maximize neuroplastic recovery (2). Robot-assisted rehabilitation is a promising therapeutic approach, enabling high-dose, repetitive, and precisely controlled training with objective performance feedback. Previous randomized controlled trials and meta-analyses have demonstrated that robotic upper limb training can improve motor function, strength, and movement quality in subacute and chronic stroke populations, supporting its clinical utility as an adjunct to conventional therapy (3–5).

Robotic rehabilitation systems can be broadly classified into end-effector–based and exoskeleton-type devices. Exoskeletal robots directly align with individual joints and provide segment-specific assistance, whereas end-effector robots interact with the distal segment and induce coordinated multi-joint movements through endpoint control (6). End-effector systems offer several practical clinical advantages, including easier setup, greater adaptability to arm length and impairment severity, and reduced risk of joint misalignment (6, 7). Moreover, by emphasizing task-oriented reaching and goal-directed movements, end-effector robots may better facilitate functional motor patterns and sensorimotor integration, particularly for chronic stroke patients with moderate-to-severe impairment (7, 8).

Beyond motor recovery, growing evidence suggests that robot-assisted training may also enhance cognitive outcomes after stroke (9). Previous studies have reported improvements in attention, executive function, visuospatial ability, and processing speed following robot-based and technology-assisted rehabilitation programs (10, 11). Interactive and feedback-driven robotic training could provide enriched cognitive engagement through real-time visual feedback, goal-oriented task execution, and error-based motor learning (12, 13). However, existing studies have had heterogeneous results, with considerable variability in intervention protocols, cognitive outcome measures, and patient populations. Additionally, most investigations have primarily focused on motor outcomes, with cognitive effects often evaluated as secondary endpoints.

Despite increasing interest in robot-assisted cognitive rehabilitation, evidence of the clinical effectiveness of end-effector–based robotic cognitive training in stroke patients remains limited. Therefore, the present study aimed to investigate the effects of end-effector robot–assisted cognitive rehabilitation on global cognitive function, detailed neuropsychological performance, and upper limb motor outcomes in patients with stroke. We hypothesized that robot-based cognitive training would measurably improve both cognitive and motor domains compared with conventional rehabilitation alone.

## Methods

### Study design and participants

This study was a single-center, randomized, assessor-blinded exploratory clinical trial designed to evaluate the effects of robot-assisted cognitive rehabilitation using the Rebless Planar system on cognitive function in patients with stroke. Participant recruitment and trial procedures were conducted from August 12, 2025 (enrollment of the first participant) to February 23, 2026 (completion of the final participant’s post-intervention evaluation). Eligible participants were adults aged ≥19 years with unilateral hemiparesis after stroke who met the predefined inclusion and exclusion criteria. Key inclusion criteria included: (1) diagnosis of stroke, (2) unilateral hemiparesis, (3) Modified Ashworth Scale ≤3 in the affected upper limb, (4) elbow Manual Muscle Test ≥2, and (5) Korean Mini-Mental State Examination (MMSE) score ≤26. Patients with severe communication disorders, significant medical or neurological comorbidities affecting training participation, severe cognitive impairment, or recent participation in another stroke-related device trial were excluded. Participants were randomly assigned in a 1:1 ratio to either the intervention or control group using a computer-generated randomization sequence with a fixed block size of 2, created by an independent researcher not involved in recruitment or outcome evaluation. To maintain allocation concealment, sequentially numbered, opaque, sealed envelopes were utilized and opened by a designated trial coordinator only after baseline clinical evaluations were completed. Outcome assessors remained strictly blinded to group allocations throughout the study period.

The sample size was determined through an *a priori* power analysis based on previous literature reporting significant MoCA improvements following conventional cognitive interventions in patients with mild cognitive impairment (14). PASS 15.0.7 software (NCSS, LLC, Utah, USA) calculations recommended a sample size of 15 participants per group to provide 80% power to detect a mean difference of 2.5 in the MoCA score (with a standard deviation of 2.3) at a two-sided significance level of 0.05.

### Intervention

**Figure 1:**
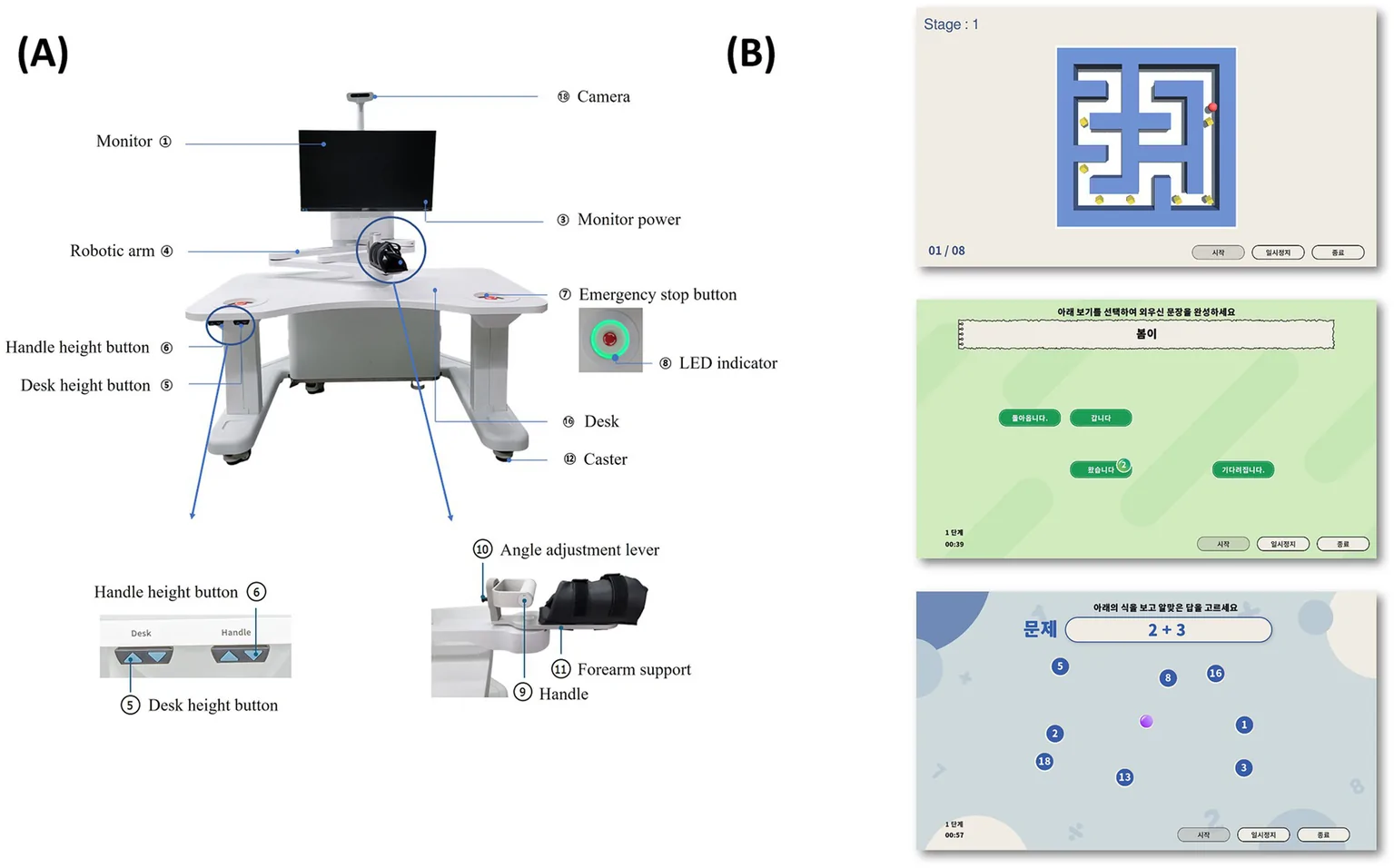
Robotic rehabilitation system and therapeutic games. **(A)** Components of the upper limb rehabilitation robot: The system includes a monitor, robotic arm, and forearm support. **(B)** Representative screenshots of therapeutic games: the cognitive-motor training programs consist of various serious games, including a Maze for spatial navigation (top), Sentence Completion for memory and linguistic processing, where the user recalls a memorized sentence and selects the correct word (middle), and a Calculation module where the user selects the correct answer by moving the handle (bottom).

Participants in the intervention group received robot-assisted cognitive rehabilitation using the Rebless Planar advanced rehabilitation program (H. Robotics Co., Ltd., Incheon, Republic of Korea). The Rebless Planar system is an end-effector–based planar robotic device that facilitates goal-directed upper extremity movements with programmable assistive and resistive forces and real-time visual feedback (Figure 1). The Rebless Planar system was previously validated in a randomized controlled trial involving patients with chronic stroke, in which robotic upper limb training with this platform led to significant improvements in motor function and spasticity, along with favorable neuroplastic changes measured by functional near-infrared spectroscopy (15).

**Figure 2:**
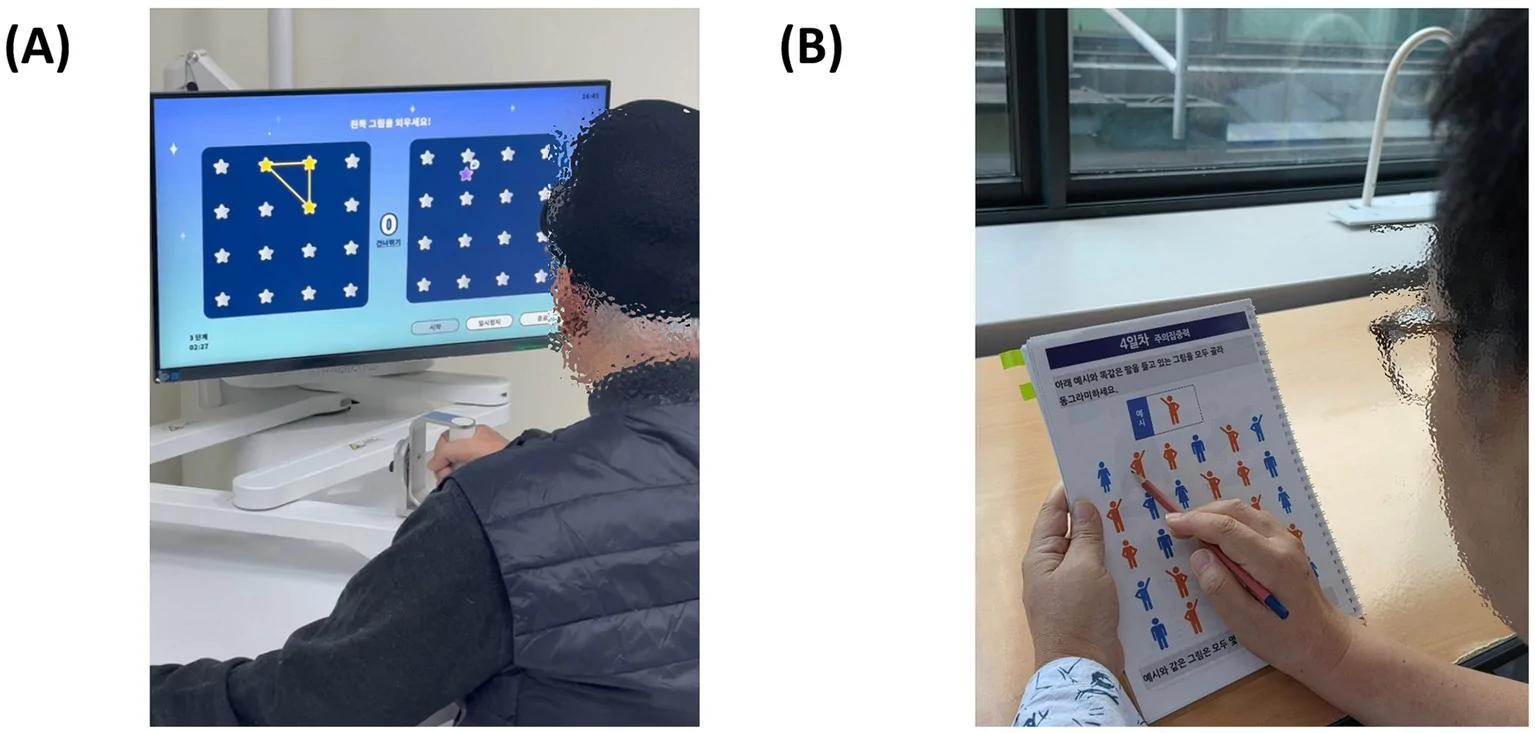
Representative intervention setups. **(A)** Intervention group: Robot-assisted integrated cognitive–motor rehabilitation using the Rebless Planar system during a goal-directed dual-task session. **(B)** Control group: Conventional paper-based cognitive rehabilitation session.

This study used the advanced cognitive training module in addition to conventional motor-based robotic exercises. This module integrates upper limb reaching and planar movement tasks with cognitively demanding activities across six specialized modules: Maze, Calculation, Sentence Completion, Orientation, Pattern Matching, and Trace Drawing. During training, participants were required to perform goal-directed arm movements to select correct visual targets, solve calculation or pattern-matching tasks, complete sentences, and respond to time- or location-based stimuli, thereby constituting an integrated cognitive–motor dual-task training paradigm (Figure 2A). Training sessions lasted 30 min, at least twice per week, for a total of 10 sessions within a three-week period. Each 30-min session consisted of a five-minute preparation phase for physical status checks and task orientation followed by a 25-min main training phase of goal-directed dual-task exercises.

The training program offered five levels of cognitive difficulty (Levels 1–5), individually tailored by adjusting the “Selection Dwell Time” (two, three, or five seconds) and the level of robotic assistance to match each participant’s functional capacity. Cognitive complexity progressively increased across levels: the Calculation module advanced from single- to double-digit operations, the Pattern Matching module increased in visual complexity, and Orientation tasks shifted from simple identification of the current date to complex temporal reasoning, such as identifying the date one week after Thanksgiving Day. To monitor intervention intensity and performance, the total number of trials and the success rate (defined as the percentage of correctly completed tasks) were automatically recorded by the robotic system. All sessions were supervised by trained rehabilitation therapists, and safety features included posture monitoring, compensatory movement feedback, and a user-accessible emergency stop mechanism. No technical malfunctions or system errors occurred throughout the trial, and the robotic system was operated in strict accordance with the safety guidelines of the Ministry of Food and Drug Safety of the Republic of Korea.

Participants in the control group received conventional paper-based cognitive rehabilitation without robotic assistance (Figure 2B). Cognitive training content was individually tailored based on each participant’s baseline neuropsychological assessment, focusing on domains identified as impaired at initial evaluation. Exercises spanned multiple cognitive domains, including orientation (e.g., identifying dates using a calendar), memory (e.g., constructing sentences with given words), attention (e.g., navigating mazes), and executive function (e.g., drawing and calculation). Tasks were systematically organized by difficulty level and adjusted to each participant’s cognitive capacity, following an errorless learning approach to maintain a high success rate and minimize frustration. Control interventions were administered for the same duration and frequency as the intervention group (30 min per session, 10 sessions over three weeks).

To ensure the objectivity of the assessments and minimize potential bias arising from the distinct natures of the robotic and paper-based interventions, a strict separation between the treating therapists and the assessors was maintained. All clinical and cognitive evaluations were performed by an independent occupational therapist who was blinded to the participants’ group assignments and was not involved in the intervention process. All assessments were conducted in a consistent environment using standardized protocols.

### Outcome measures

Global cognitive function, the primary outcome measure, was assessed using the MMSE and the Korean version of the Montreal Cognitive Assessment (MoCA). The MMSE is a widely used screening tool for global cognitive impairment, evaluating orientation, attention, memory, language, and visuospatial function. The MoCA is considered more sensitive for detecting mild cognitive impairment, particularly in executive and attentional domains frequently affected after stroke (16).

Secondary outcome measures included a comprehensive neuropsychological assessment using the Korean version of the Consortium to Establish a Registry for Alzheimer’s Disease (CERAD) battery, which evaluates multiple cognitive domains commonly impaired in cerebrovascular disease (17). The CERAD subdomains assessed in this study included language function, confrontation naming as measured by the Boston Naming Test, verbal learning and memory (word list memory, recall, and recognition), and visuoconstructive ability and memory (constructional praxis and recall). Executive function and processing speed were evaluated as part of the CERAD battery using the Trail Making Test parts A and B (TMT-A and TMT-B), which assess visual attention, psychomotor speed, and cognitive flexibility. Additionally, inhibitory control and selective attention were assessed using the Stroop Word, Color, and Color–Word tests, which are established CERAD components reflecting frontal-executive dysfunction.

Upper extremity motor function was assessed using the Fugl–Meyer Assessment for the Upper Extremity (FMA-UE), a validated and stroke-specific measure of motor impairment. Functional independence in activities of daily living was evaluated using the Modified Barthel Index (MBI), which reflects overall disability and daily functional performance. Additionally, participants in the intervention group completed a satisfaction questionnaire after the intervention to assess usability and perceived benefits of Rebless Planar training. The questionnaire consisted of 12 items, each rated on a 1–4 Likert scale.

### Statistical analysis

Baseline demographic and clinical characteristics were compared between groups using independent t-tests or chi-square tests, as appropriate. Within-group changes from pre- to post-intervention were analyzed using paired t-tests. To account for any potential baseline imbalances, between-group differences in outcome changes were assessed using analysis of covariance, with baseline scores included as a covariate. Because all 30 randomized participants completed the full 10-session intervention protocol and post-intervention evaluations, no data imputation procedures for missing observations were necessary. To control for Type I error inflation across the secondary cognitive subdomains (CERAD battery), *p*-values were adjusted using the Benjamini–Hochberg False Discovery Rate (FDR) procedure. Effect sizes were calculated as Cohen’s *d* for between-group change differences. Statistical significance was set at *p* < 0.05. Data analysis was performed using R 4.5.0 (R Foundation for Statistical Computing, Vienna, Austria).

## Results

**Figure 3:**
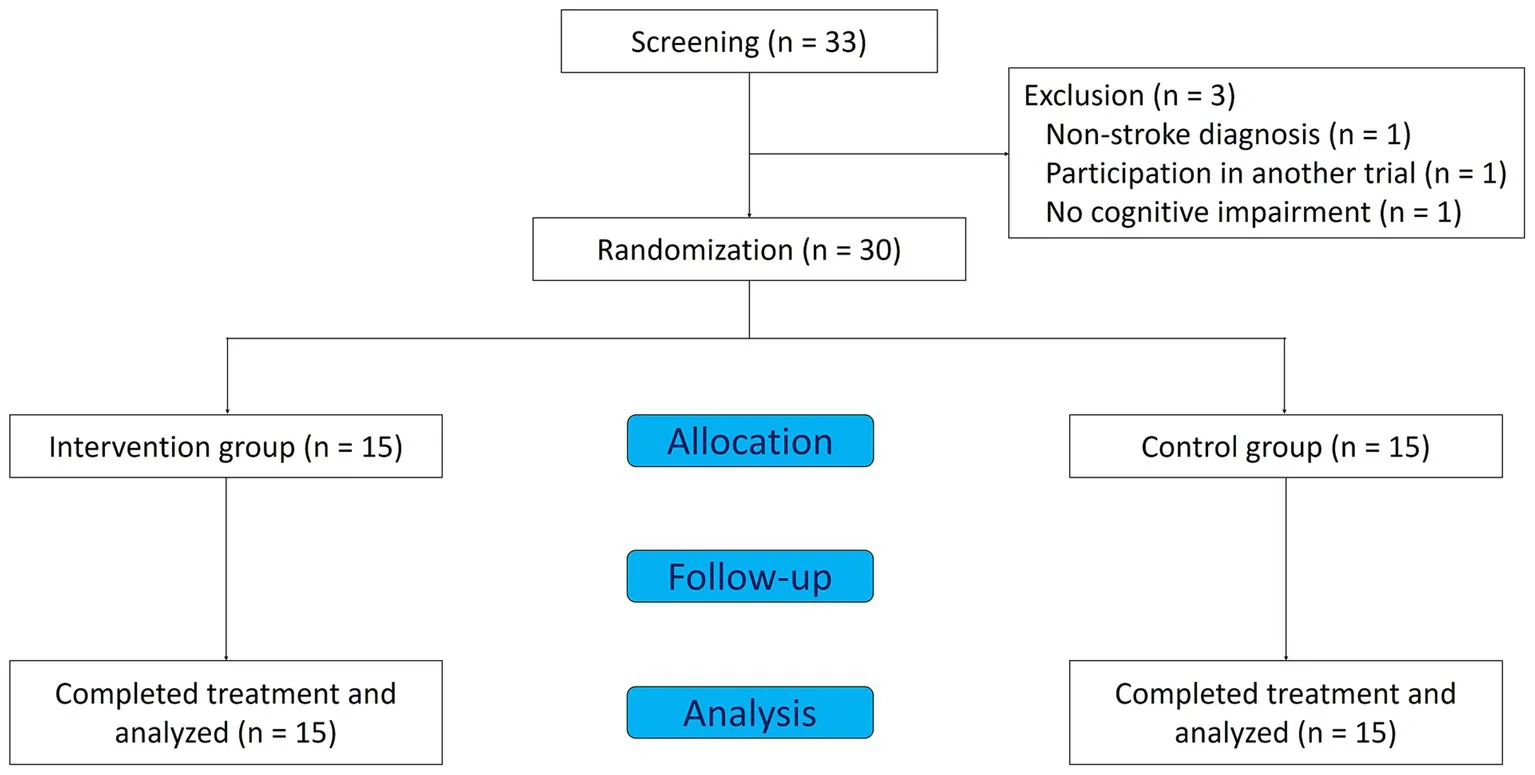
CONSORT flow diagram of the study.

Thirty participants were included in the final analysis (Figure 3). Baseline demographic and clinical characteristics were comparable between the two groups, with no significant differences in age, sex distribution, body mass index, time since stroke onset, stroke type, or stroke laterality (Table 1).

**Table 1:** Demographic data.

Variables	Intervention (*n* = 15)	Control (*n* = 15)	*p*-value
Age	68.2 ± 7.8	67.6 ± 9.9	0.854
Gender			1.000
Male	6 (40.0%)	6 (40.0%)	
Female	9 (60.0%)	9 (60.0%)	
Body mass index	23.9 ± 3.0	24.0 ± 3.1	0.840
Education years	11.4 ± 3.8	11.0 ± 3.3	0.759
Stroke onset, month	167.3 ± 60.6	163.3 ± 61.6	0.726
Stroke type			0.449
Infarction	11 (73.3%)	8 (53.3%)	
Hemorrhage	4 (26.7%)	7 (46.7%)	
Stroke laterality			0.680
Right	12 (80.0%)	10 (66.7%)	
Left	3 (20.0%)	5 (33.3%)	

**Figure 4:**
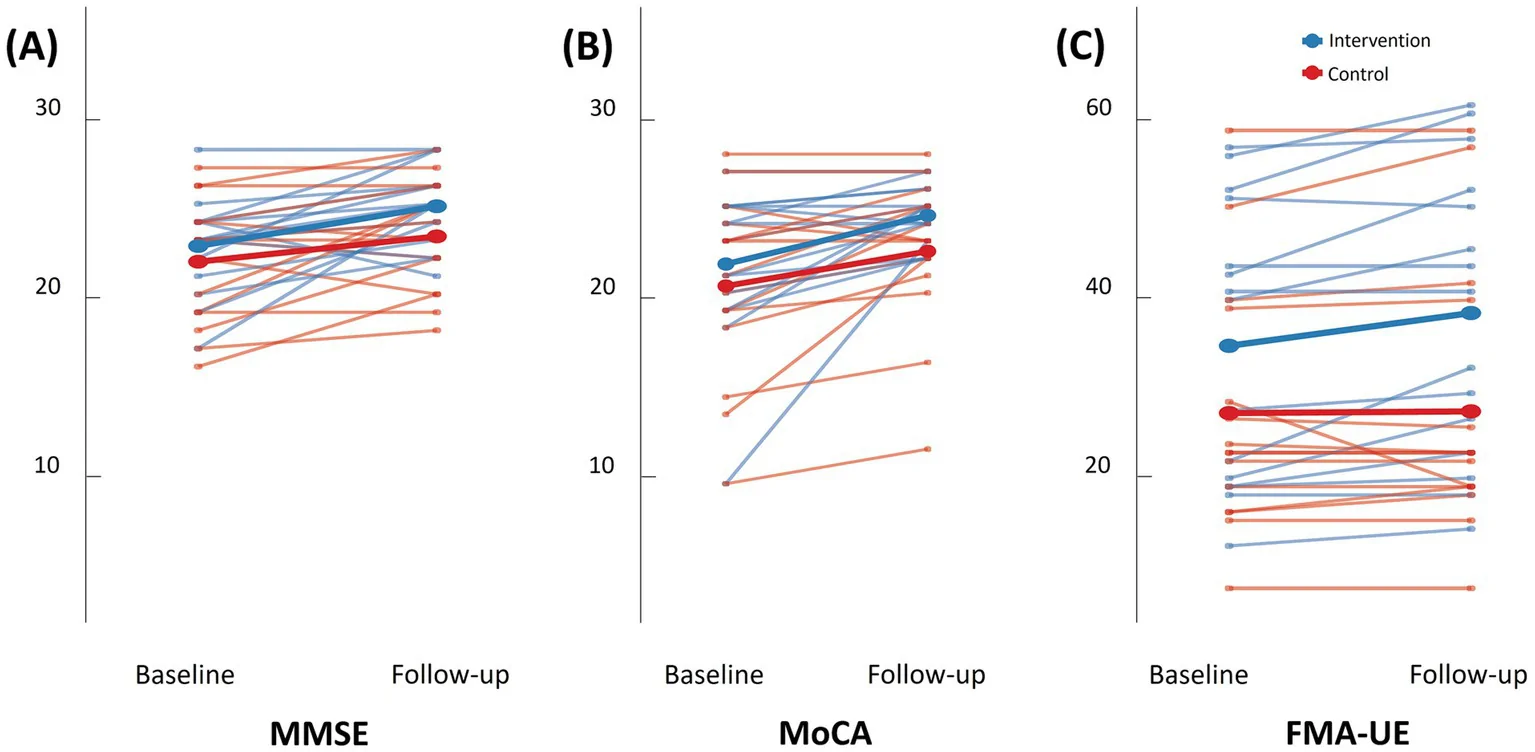
Spaghetti plots for clinical outcomes. Plots illustrate individual (light lines) and mean (bold lines) changes from baseline to follow-up for **(A)** Mini-Mental State Examination (MMSE), **(B)** Montreal Cognitive Assessment (MoCA), and **(C)** Fugl-Meyer Assessment of the Upper Extremity (FMA-UE). The intervention group is represented by blue lines, and the control group is in red.

The intervention group exhibited significant improvements in global cognitive function following robot-assisted cognitive rehabilitation. Both MMSE (*p* = 0.008) and MoCA (*p* = 0.013) scores increased significantly from baseline. The control group also demonstrated significant improvements in MMSE (*p* = 0.039) and MoCA (*p* = 0.012) (Table 2). However, no significant between-group differences were detected for either MMSE (*p* = 0.403) or MoCA (*p* = 0.512), as illustrated by the overlapping trajectories in the spaghetti plots (Figures 4A,B).

**Table 2:** Changes in functional outcomes.

Variables	Intervention (*n* = 15)			Control (*n* = 15)		*p*-value	Effect size	Between group *p*-value
	Pre	Post	*p*-value	Pre	Post			
MMSE	24.7 ± 2.6	26.9 ± 2.2	0.008*	23.8 ± 3.4	25.2 ± 3.0	0.039*	0.31	0.403
MoCA	21.7 ± 4.5	24.5 ± 1.7	0.013*	20.4 ± 5.3	22.4 ± 4.3	0.012*	0.24	0.512
CERAD								
Language	10.7 ± 3.2	11.1 ± 2.3	0.651	12.3 ± 4.9	13.5 ± 5.3	0.129	−0.26	0.852
Boston naming test	11.5 ± 1.8	12.4 ± 1.1	0.038*	11.3 ± 2.5	11.7 ± 2.5	0.313	0.42	0.852
Word list memory	16.1 ± 3.7	20.0 ± 3.7	<0.001*	14.4 ± 4.9	16.5 ± 5.6	0.002*	0.74	0.413
Word list recall	5.2 ± 1.3	6.6 ± 1.4	<0.001*	3.6 ± 2.4	4.9 ± 2.4	0.006*	0.05	1.000
Word list recognition	9.1 ± 0.9	9.2 ± 0.9	0.827	8.3 ± 1.8	8.3 ± 2.0	0.843	0.00	1.000
Constructional praxis	10.3 ± 1.2	10.4 ± 1.2	0.737	9.3 ± 1.5	9.9 ± 1.2	0.164	−0.33	0.852
Constructional recall	6.3 ± 2.3	7.9 ± 2.4	0.024*	5.8 ± 3.6	6.6 ± 4.6	0.267	0.33	0.852
Trail making test A	81.7 ± 63.1	61.9 ± 34.5	0.031*	80.9 ± 36.4	69.0 ± 30.7	0.019*	−0.31	0.852
Trail making test B	178.9 ± 45.6	173.0 ± 55.3	0.587	212.0 ± 82.9	210.5 ± 87.8	0.843	−0.13	0.904
Stroop test: word	49.4 ± 17.9	52.5 ± 17.4	0.111	44.3 ± 24.0	49.1 ± 26.2	0.083	−0.20	0.852
Stroop test: color	35.8 ± 18.0	39.4 ± 19.7	0.069	32.3 ± 26.5	38.3 ± 25.5	0.151	−0.20	0.852
Stroop test: color-word	21.9 ± 12.7	24.5 ± 13.9	0.138	17.8 ± 16.9	22.1 ± 17.0	0.206	−0.17	0.865
FMA-UE	33.6 ± 16.5	37.5 ± 17.1	0.002*	25.7 ± 14.8	25.9 ± 15.8	0.826	0.98	0.012*
MBI	89.3 ± 6.8	90.8 ± 6.7	0.140	84.6 ± 10.1	86.2 ± 9.5	0.081	−0.02	0.959

*p-value < 0.05.

Values are presented as mean ± standard deviation. Between-group *p*-values for CERAD subdomains were adjusted for multiple testing using the Benjamini–Hochberg False Discovery Rate procedure. Effect size is reported as Cohen’s d for between-group change differences.

FMA-UE, Fugl-Meyer Assessment of Upper Extremity; MBI, Modified Barthel Index; MMSE, Mini-Mental State Examination; MoCA, Montreal Cognitive Assessment.

Regarding secondary CERAD subdomains, significant within-group improvements were observed in several domains for both groups, including word list memory, word list recall, and TMT-A (all within-group *p* < 0.05) (Table 2). However, no statistically significant between-group differences were detected across any CERAD subdomains after FDR adjustment.

Upper extremity motor function, assessed using the FMA-UE, improved significantly in the intervention group (*p* = 0.002), whereas no significant change was observed in the control group (*p* = 0.826). The between-group difference for FMA-UE was statistically significant (*p* = 0.012), indicating greater motor improvement in the intervention group. This distinct improvement is further supported by the spaghetti plot (Figure 4C), which exhibits a clearer upward trend in the intervention group compared to the control group. Functional independence, assessed using MBI, did not change significantly in the intervention group (*p* = 0.140) or the control group (*p* = 0.081), and no significant between-group difference was observed (*p* = 0.959).

Finally, the intervention group (*n* = 15) was given a 12-item questionnaire assessing participant satisfaction and the usability of the robot-assisted therapy and perceived effectiveness. Overall satisfaction was high across domains, ranging from 3.20 to 3.73 on the 4-point Likert scale. Regarding the intervention intensity, the participants in the intervention group performed an average of 525.8 ± 63.9 trials throughout the study period. The overall success rate of these robot-assisted tasks was 82.9%. No adverse events were reported during the study, and all participants completed the intervention and follow-up assessments.

## Discussion

In this prospective exploratory randomized controlled trial, robot-assisted integrated cognitive-motor rehabilitation demonstrated feasibility, safety, and superior efficacy in upper limb motor recovery compared to conventional cognitive training in chronic stroke patients. Although both groups exhibited within-group improvements in global cognitive measures (MMSE and MoCA), no significant between-group differences were observed in cognitive outcomes. Consequently, the primary hypothesis of superior cognitive enhancement through the robot-assisted dual-task intervention was not confirmed. Nevertheless, the intervention group showed clinically meaningful and statistically significant improvements in upper extremity motor function (FMA-UE) that were not observed in the control group, highlighting the potential utility of end-effector robotic platforms for motor rehabilitation. Additionally, participants in the intervention group reported high levels of usability and overall satisfaction, with no adverse events reported during the study, supporting the safety and practical feasibility of incorporating integrated cognitive–motor protocols into clinical stroke rehabilitation.

A strength of the Rebless Planar system is its capacity to host intelligent feedback games that naturally integrate cognitive challenges with physical movements. This integration is based on the dual-task paradigm, which requires the simultaneous allocation of cognitive resources (e.g., attention, memory, executive control) and motor resources (18). Regarding secondary cognitive outcomes, participants in the intervention group exhibited significant within-group improvements across several CERAD subdomains, including the Boston Naming Test, word list memory, word list recall, constructional recall, and TMT-A. However, these improvements were largely based on within-group changes rather than statistically significant between-group differences. Furthermore, because the control group received an active, time-matched paper-based cognitive rehabilitation program, the absence of significant between-group differences in cognitive measures was methodologically expected, as both cohorts engaged in structured cognitive stimulation. These subdomain changes should be regarded as exploratory observations that suggest potential benefits in verbal memory and visual processing speed. Theoretically, such dual-task reaching requires multi-sensory encoding—combining the auditory word, visual image, and physical reaching movement—which may help reinforce memory traces within the hippocampal-prefrontal network (19). Additionally, the interactive, gamified nature of the Rebless Planar provides immediate feedback, which maintains high levels of arousal and attention. This heightened attention is a prerequisite for “encoding” information into long-term memory. When a patient is motivated by the reward of game progress, they are more likely to successfully store and subsequently recall the target information (20).

End-effector systems, such as the Rebless Planar, interface with the patient at a single distal point to guide movement of the entire limb. This distal drive mechanism allows for a more natural proximal joint coordination, as the shoulder and elbow are free to move in a self-organized manner to achieve a specific spatial trajectory. This flexibility significantly reduces setup time and enhances the device’s utility in diverse clinical settings, as it can be quickly adjusted to patients of varying body types without the need for meticulous joint-by-joint alignment (7). Meta-analytical evidence indicates that end-effector robots are often superior to exoskeletons in improving motor control across all upper limb joints, particularly in patients with moderate to severe stroke. This is attributed to end-effector systems’ inherent free coordination, which forces the brain to actively plan and execute multi-joint synergies rather than passively following a prescribed joint-specific path (3). Consistent with these findings, our study observed a significant improvement in FMA-UE scores within the intervention group (+3.9 points, *p* = 0.002), which closely approaches the lower threshold of the minimal clinically important difference reported as 4.25–7.25 points in chronic stroke (21). These results align with a previous study using the Rebless Planar system, which demonstrated significant motor recovery and favorable neuroplastic changes. Notably, that study utilized functional near-infrared spectroscopy and reported a significant decrease in the unaffected hemisphere’s cortical activity alongside improved motor performance, suggesting a shift toward more balanced interhemispheric inhibition and organized motor control (15).

Several limitations of this study should be acknowledged. First, this was a small-scale randomized controlled trial conducted at a single center, which may limit the generalizability of the findings. Although randomization was performed, residual and unmeasured confounding factors could have influenced participants’ differing recovery trajectories. For instance, pre-stroke cognitive reserve and specific lesion characteristics were not fully analyzed. These factors may play a role in how individual patients respond to integrated rehabilitation interventions. Second, the study evaluated only short-term effects immediately following the intervention period. As cognitive recovery after stroke is a dynamic and prolonged process, the lack of long-term follow-up precludes conclusions regarding the durability and sustainability of the observed cognitive and motor improvements. Third, although the intervention was designed as an integrated cognitive–motor training paradigm, mechanistic insights into the neural processes underlying the observed improvements could not be explored. Neurophysiological and neuroimaging measures, such as electroencephalography or functional near-infrared spectroscopy, were not included. Therefore, changes in cortical activation patterns, network connectivity, or neuroplasticity associated with the intervention remain speculative. Fourth, a methodological limitation regarding the control group design should be noted. Although both interventions were time- and dose-matched, the control group received paper-based cognitive rehabilitation without an active motor component. Consequently, the superior FMA-UE gains in the intervention group cannot be conclusively attributed solely to the robotic system itself. Nevertheless, this outcome highlights that within the same allocated therapy timeframe, integrating motor reaching into cognitive rehabilitation provides dual-domain benefits without compromising cognitive engagement. Fifth, although the sample size was powered based on an expected difference in MoCA scores, the primary endpoint was not achieved, indicating that the trial may have been underpowered to detect subtle between-group cognitive differences. Consequently, secondary findings (such as FMA-UE motor gains) must be interpreted strictly as exploratory observations and cannot substitute for an unconfirmed primary endpoint to claim overall intervention efficacy.

## Conclusion

This exploratory randomized controlled trial demonstrates that robot-assisted integrated cognitive–motor rehabilitation using the end-effector Rebless Planar system is safe and clinically feasible for chronic stroke patients. Although the intervention led to positive within-group trends in cognitive performance, it did not demonstrate statistically significant superiority over conventional paper-based cognitive rehabilitation in improving global cognitive function (MMSE and MoCA). Notably, however, the intervention group exhibited superior upper limb motor recovery (FMA-UE) compared to the control group, suggesting that goal-directed dual-task robotic training is a useful strategy primarily for motor enhancement. Future large-scale trials with adequately powered samples, longer follow-up periods (e.g., at 1 and 3 months post-intervention), and neurophysiological evaluations are needed to clarify the specific cognitive impacts and underlying neural mechanisms of this integrated approach.

## Data Availability

The raw data supporting the conclusions of this article will be made available by the authors, without undue reservation.
