# Supplementary material for: Robot-assisted integrated cognitive-motor rehabilitation in patients with chronic stroke: a randomized controlled trial
Source: Front Neurol. 2026 Aug 13;17:1866135. doi: 10.3389/fneur.2026.1866135 (PMC13518322; doi:10.3389/fneur.2026.1866135)
Supplement: Supplementary file 1 [file Supplementary_file_1.docx]

**Supplementary Table S1.** User satisfaction questionnaire. The questionnaire was administered to the 15 participants in the intervention group. All responses were rated on a 1–4 Likert scale, where 1 = strongly disagree, 2 = disagree, 3 = agree, and 4 = strongly agree.

| Classification | Questions | Mean ± Standard deviation |
| --- | --- | --- |
| Usability | Was the operation of this medical device easy to use? | 3.20 ± 0.77 |
|  | Did you feel no discomfort or dissatisfaction during the training? | 3.47 ± 0.92 |
| Perceived Effectiveness | Did you feel that the cognitive rehabilitation program of this medical device was effective? | 3.60 ± 0.83 |
|  | Did you feel that the 'Maze' program was effective for cognitive rehabilitation? | 3.73 ± 0.59 |
|  | Did you feel that the 'Calculation' program was effective for cognitive rehabilitation? | 3.53 ± 0.83 |
|  | Did you feel that the 'Sentence Completion' program was effective for cognitive rehabilitation? | 3.73 ± 0.80 |
|  | Did you feel that the ‘Orientation’ program was effective for cognitive rehabilitation? | 3.67 ± 0.49 |
|  | Did you feel that the 'Pattern Matching' program was effective for cognitive rehabilitation? | 3.67 ± 0.62 |
|  | Did you feel that the 'Trace Drawing' program was effective for cognitive rehabilitation? | 3.60 ± 0.63 |
|  | Do you believe that rehabilitation training using this device helps improve cognitive function? | 3.67 ± 0.62 |
| Overall Satisfaction | Were you satisfied with your overall experience using this medical device? | 3.73 ± 0.59 |
|  | Do you have the intention to continue receiving robot-assisted rehabilitation after the study? | 3.67 ± 0.82 |
